# Nuclear Molecular Imaging Strategies in Immune Checkpoint Inhibitor Therapy

**DOI:** 10.3390/diagnostics7020023

**Published:** 2017-04-21

**Authors:** Kasper F. Guldbrandsen, Helle W. Hendel, Seppo W. Langer, Barbara M. Fischer

**Affiliations:** 1 Department of Pulmonary and Infectious Diseases, Nordsjællands Hospital Hillerød, 3400 Hillerød, Denmark; kasper.foged.guldbrandsen.01@regionh.dk; 2 Department of Clinical Physiology and Nuclear Medicine, Herlev and Gentofte Hospital, 2750 Herlev, Denmark; helle.westergren.hendel@regionh.dk; 3 Department of Oncology 5073, Rigshospitalet, 2100 Copenhagen, Denmark; seppo.langer@regionh.dk; 4 Department of Clinical Physiology, Nuclear Medicine and PET, Rigshospitalet, 2100 Copenhagen, Denmark

**Keywords:** immune checkpoint inhibitor therapy, PET/CT, radiotracer, response evaluation/treatment monitoring

## Abstract

Immune checkpoint inhibitor therapy (ICT) is a new treatment strategy developed for the treatment of cancer. ICT inhibits pathways known to downregulate the innate immune response to cancer cells. These drugs have been shown to be effective in the treatment of a variety of cancers, including metastatic melanoma and lung cancer. Challenges in response evaluation of patients in ICT have risen as immune related side effects and immune cell infiltration may be confused with progressive disease. Furthermore, the timing of the evaluation scan may be challenged by relatively slow responses. To overcome this, new response criteria for evaluating these patients with morphologic imaging have been proposed. The aim of this paper is to review and discuss the current evidence for the use of molecular imaging, e.g., PET/CT (Positron Emission Tomography/Computer Tomography) with ^18^F-Fluorodeoxyglucoes (FDG) as an alternative imaging method for monitoring patients undergoing ICT. Following the currently available evidence, this review will primarily focus on patients with malignant melanoma.

## 1. Introduction

Evasion of attack by the immune system is one of the hallmarks of cancer [1]. Based on the amount of mutations being accumulated by cancer cells, immune cells should register cancer cells as being foreign. One reason why this fails is because of the development of tolerance.

Several mechanisms guide the development of tolerance, including immune checkpoint pathways, which downregulates immune function to prevent immune cells from being self-reactive. Blocking these immune checkpoints stimulates an autoimmune response, but also help immune cells recognizing foreign cells such as cancer.

Drugs that inhibit immune checkpoints have shown clinical efficacy in several different types of cancer, e.g., melanoma, non-small cell lung cancer (NSCLC), renal and urothelial cancer, Hodgkin’s lymphoma and head- and neck cancer [2,3,4,5,6,7]. Encouraging results have been demonstrated especially in advanced melanoma for which the previous therapeutic options have been limited, as well as in metastatic NSCLC, currently replacing traditional platin-based chemotherapy as first-line therapy in selected patients [8,9].

The effect of immune checkpoint inhibitor therapy (ICT) on cancer cells is mediated thru activation of an immune response against the cancer cells. This indirect, immune-mediated effect has given rise to new challenges concerning response evaluation as response patterns to ICT differ from those observed with conventional anti-cancer therapy. The aim of this article is to provide an overview of the challenges and possibilities for molecular imaging, such as positron emission tomography (PET) and single-photon emission computed tomography (SPECT), in the evaluation of patients treated with ICT. In order to put the use of different imaging modalities into the right perspective, this review starts with a brief introduction to ICT and examples of current use in patients with malignant melanoma and NSCLC.

## 2. Immune Checkpoint Therapy

The current ICT is based on regulation of two important immune checkpoint pathways: one involving programmed cell death protein (PD-1/PD-L1) and one involving the cytotoxic T-lymphocyte-associated protein 4 (CTLA-4).

Programmed cell death protein 1 (PD-1), is a cell surface receptor that is expressed on T cells, B cells, natural killer T cells, activated monocytes, and dendritic cells [10]. T-cells require two signals to become activated. First, a signal triggered by the interaction between antigen-major histocompatibility complex (MHC) and the T cell receptor, and second a co-stimulatory signal provided by antigen-presenting cells (APCs). T-cell activation causes T cell clonal expansion, cytokine secretion and the effector function of the T cell. Binding of PD-1 to its ligand PD-L1 inhibits the co-stimulatory signal, causing a decrease in the production of cytokines and cell survival proteins, ultimately downregulating the activity of self-reactive T-cells. PD-L1 is expressed by normal immune cells as well as by many different tumor types, allowing the tumor cells to evade the immune system.

The anti-PD-1 antibodies pembrolizumab and nivolumab have shown promising results in patients with metastatic melanoma not harboring the mutation in the B-Raf proto-oncogene (BRAF-mutation) with improved survival rates when compared to the anti CTLA-4 antibody ipilimumab and chemotherapy [11,12,13]. Response has been proved durable with two-year survival rates of 43% [14]. Pembrolizumab has also been shown to be an effective treatment option in advanced NSCLC, with an overall survival of 17.3 months, compared to 8.2 months with docetaxel [3].

Another important immune checkpoint involves CTLA-4. Structurally related to the CD28 receptor, it is primarily expressed on the surface of T cells. CD28 mediates the co-stimulatory signal from APCs required for activation of T cell effector function, interacting with B7-1 and B7-2 on APCs. This effect is caused by CTLA-4 ligating with B7-1 and B7-2, resulting in a downregulatory signal.

One CTLA-4 antibody, ipilimumab, has been shown in multiple phase II/III trials to be an effective therapeutic option in the treatment of metastatic melanoma. In previously untreated patients with metastatic melanoma, the combination of ipilimumab and chemotherapy was more effective than that of chemotherapy alone, with more than double the number of long-term survivors in the ipilimumab group [8,15].

A disadvantage of therapies targeting immune checkpoint pathways is the increased rate of immune-related adverse events (irAEs), resulting from the lowered inhibition of T cell activity, thereby causing self-reactivity. Because the development irAEs are caused by increased T cell reactivity, these reactions could potentially be correlated with increased anti-tumor activity, although this effect is likely offset by discontinuation of therapy when irAEs arise.

Most reported irAEs are diarrhea, pruritus, rash, colitis vitiligo and endocrine irAEs such as thyroiditis and hypophysitis. More rarely seen are rheumatic and granulomatous irAEs such as arthritis and sarcoid-like disease. In studies with the CTLA-4 inhibitor, ipilimumab, the frequency of grade 3 or 4 irAEs is reported in the range of 10% to 43%: with studies using a dose of 10 mg/kg of ipilimumab having a higher incidence of irAEs than studies with a dose of 3 mg/kg [2,15,16]. Compared to ipilimumab, the PD-1 inhibitor, pembrolizumab, has been shown to have fewer treatment related adverse events[11], and severe adverse events has been shown to be less common with pembrolizumab than with docetaxel [3].

## 3. Patterns of Response

With conventional treatment modalities in cancer therapy such as chemotherapy, tumor cells are being affected directly by the drug, inhibiting mitosis and blocking extracellular growth signals, causing cell death, tumor necrosis and inhibition of tumor growth.

In contrast to this, therapies targeting immune checkpoint pathways affect cancer cells indirectly through mobilizing an immune response against the cancer cells, causing infiltration of inflammatory cells into the tumor environment, followed by inflammation and cytotoxicity. In practice, this causes response patterns during ICT that differ from those seen during chemotherapy, e.g., delayed response to treatment, or response preceded by transient worsening (so-called pseudoprogression) [17,18,19,20].

CTLA-4 blockade has been shown to frequently induce intratumoral infiltration by immune cells, in both clinical responders and non-responders, potentially causing pseudoprogression as can be seen in early and sometimes also late scans in patients undergoing ICT [21,22]. The apparent worsening can be seen as increased size of known lesions or the appearance of new ones. Trials with ipilimumab in melanoma patients have found that onset of partial response (PR) or complete response (CR) occurs after >12 weeks of treatment in up to 68% of patients with objective response (OR) [23]. Four distinct patterns of response have been described in these patients: (1) shrinkage in baseline lesions; (2) durable stable disease (sometimes followed by slow steady decline in tumor burden); (3) response after initial disease progression/transient worsening (pseudo-progression) and (4) simultaneous response in baseline lesions and presence of new lesions [24]. These patterns of response challenge the current Response Evaluation Criteria in Solid Tumors (RECIST) as the standard response criteria in trials having tumor response as endpoint [25]. In RECIST, tumor response is defined by changes in overall tumor burden from baseline measurements and the appearance of new lesions. According to RECIST, both response after initial disease progression and response in the presence of new lesions would be classified as progressive disease (PD).

### Immune-Related Response Criteria (irRC)

Experience from the evaluation of cancer vaccines, where the effect on tumor cells is mediated through immune activation similar to immune checkpoint inhibitors, led to the first proposal of immune-related response criteria (irRC) in 2009. It was based on the WHO-criteria (bi-dimensional measurement of target lesion) for response evaluation [24]. In 2013, a revised irRC were published using uni-dimensional measurements [26]. The main difference between irRC and WHO as well as RECIST is the incorporation of new measurable lesions into total tumor burden instead of always representing progressive disease (PD) and that PD is defined as more than a 25% increase in total tumor burden compared with nadir, in two consecutive observations at least four weeks apart.

With these new response criteria, it has been demonstrated that among patients who received ipilimumab and were initially classified as having PD at week 12, 22 out of 227 patients had a response to therapy according to irRC [24]. Similarly, in a trial of melanoma patients receiving pembrolizumab therapy, 14% (84/592) of patients, who were initially classified as having PD according to RECIST v1.1, had non-progressive (non-PD) disease per irRC and the two-year survival rate was higher for patients having PD per RECIST v1.1 criteria and non-PD per irRC (37.5%) than for patients having PD per both criteria (17.3%) [20]. These results suggest that adapting therapy response criteria to the new response patterns seen with ICT in melanoma patients may improve the accuracy of response evaluation. Though these adapted criteria avoid discontinuing treatment in patients with delayed response or pseudoprogression, this also increases the amount of patients receiving ineffective treatment.

In March 2017, the RECIST working group suggested new guidelines for the collection of data in trials testing immunotherapeutics iRECIST [27]. Based on RECIST 1.1, it introduces a new response designation iUPD (unconfirmed progressive disease, the “i” designating the immunotherapeutic setting). If iUPD is confirmed on subsequent scans by a further increase in size of the lesion, response is designated iCPD (confirmed progressive disease). If not confirmed, response is designated as iCR, iPR or iSD and status is reset, so any increase in size of lesions in subsequent assessments is again designated iUPD. The revised guidelines also suggest that continuation of treatment in patients with iUPD should only be done if a patient is clinically stable and that the next assessment should be done within eight weeks so that patients remain candidates for alternative therapeutic strategies.

## 4. FDG PET/CT

As an alternative to anatomical imaging, molecular imaging modalities such as Positron Emission Tomography/Computer Tomography with ^18^F-Fluorodeoxyglucose (FDG PET/CT) has been suggested as a tool for faster and more accurate response evaluation during ICT. Malignant melanoma is a highly FDG-avid cancer and FDG PET/CT has been shown to be superior to CT in the detection of distant metastases and in recurrence [28,29,30,31]. FDG PET/CT has been successfully used in early response evaluation of conventional chemotherapy in different non-melanoma cancers. Thus, FDG PET/CT could be a promising alternative to CT for monitoring treatment response in patients undergoing ICT. The evidence, which we will review in the following, is however still scarce. An overview of the results is presented in Table 1.

Sachpekidis and colleagues found that FDG PET scans evaluated using European Organization for Research and Treatment of Cancer (EORTC) criteria after two cycles of ipilimumab in patients with metastatic melanoma was predictive of final treatment response in 18 of the 22 patients enrolled. Two patients were initially classified as having stable metabolic disease (SMD), while the late scan (after four cycles) showed progressive metabolic disease (PMD), and two patients, who ended up having partial metabolic response (PMR) on the late scan, were originally wrongly classified as having PMD on the early scan [32]. Similarly, a study from Australia in 27 melanoma patients undergoing prolonged treatment with a PD-1 inhibitor found that, amongst those with residual disease on CT, 43% had negative FDG PET scans and that none of the patients with negative FDG PET scans progressed within the 6–10 months follow-up period [33].

In pre- and post-treatment, FDG PET-CT scans of melanoma patients undergoing treatment with ipilimumab, fractal and multifractal analyses showed potential as a non-operator dependent biomarker for treatment response monitoring [34]. The algorithms used did not, however, account for physiological uptake of the tracer in organs such as in the brain nor non-malignant local increases in tracer uptake associated with irAEs.

In an abstract pertaining to a retrospective study of 28 melanoma patients treated with ipilimumab who had follow-up FDG PET/CT after 2–4 cycles of treatment, PMD was highly correlated with clinical progressive disease with a positive predictive value (PPV) of 93% [35]. Two-year survival rates were 31% for patients with PMD and 73% for patients with non-PMD. These preliminary survival data on responders and non-responders are similar to those seen when evaluating treatment response using CT with irRC or RECIST [20]. In another abstract, results from a phase II study of atezolizumab (a PD-L1 inhibitor) in 138 stage IIIB/IV NSCLC patients are presented [36]. These patients underwent FDG PET/CT at baseline and at week 6 of treatment. This study found that patients with metabolic response on PET/CT at week 6 had higher ORR (overall response rate) than metabolic non-responders (73.9% vs. 6.3%).

It appears that, although response evaluation using FDG PET/CT misclassifies some after two cycles, evaluation of treatment response after four cycles using FDG PET/CT has similar accuracy in treatment response evaluation as using irRC based on CT. Thus, the potential ability of FDG-PET to diagnose response early, combined with the ability to discriminate between active and inactive residual disease on CT and an increased efficacy in the detection of metastasis could prove FDG PET/CT superior in the long term for monitoring of patients undergoing ICT. There are currently ongoing trials investigating FDG PET/CT response monitoring in metastatic melanoma patients treated with pembrolizumab and nivolumab [37,38].

### Pseudoprogression

**Figure 1:**
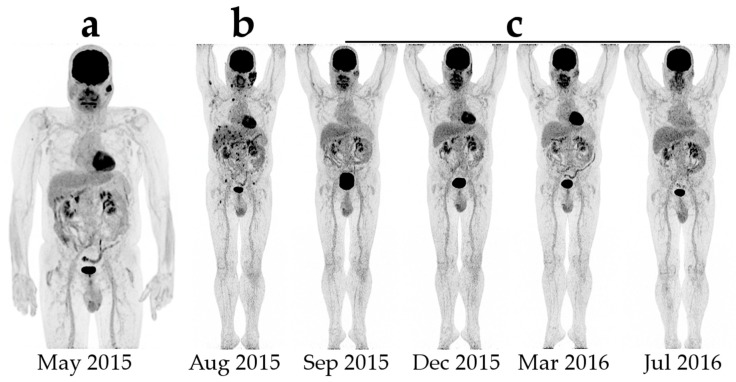
Example of early pseudoprogression in a patient with malignant melanoma: (**a**) initial Positron Emission Tomography/Computer Tomography with ^18^F-Fluorodeoxyglucose; (**b**) first evaluation after four series of ipilimumab shows multiple new foci in the liver and the bones with highly increased LDH (lactate dehydrogenase); and (**c**) subsequent scans after completion of treatment showing complete disappearance of both new foci and the primary lesions.

Pseudoprogression is seen on both CT and FDG-PET as a prematurely diagnoses of progressive disease (Figure 1). The apparent progression is both caused by the invasion of immune cells into the tumors causing the tumors to increase in size and to accumulate FDG and the continued tumor growth during therapy, until a sufficient immune response is developed [24]. Using irRC, two types of pseudoprogression has been described: (1) early pseudoprogression with more than 25% increase in tumor burden at 12 weeks and not confirmed as PD at the next assessment; and (2) late pseudoprogression with more than 25% increase in tumor burden after 12 weeks that was not confirmed as PD at the next assessment. Early pseudoprogression is probably most common. In the new iRECIST, both scenarios are classified as iUPD, and a patient can have several instances of iUPD, but not iCPD, before response (iCR, iPR or iSD) [27]. It is important to stress that pseudoprogression is a relatively rare phenomenon seen only in 3%–10% of the patients and that it is a retrospective diagnosis [20,24].

**Figure 2:**
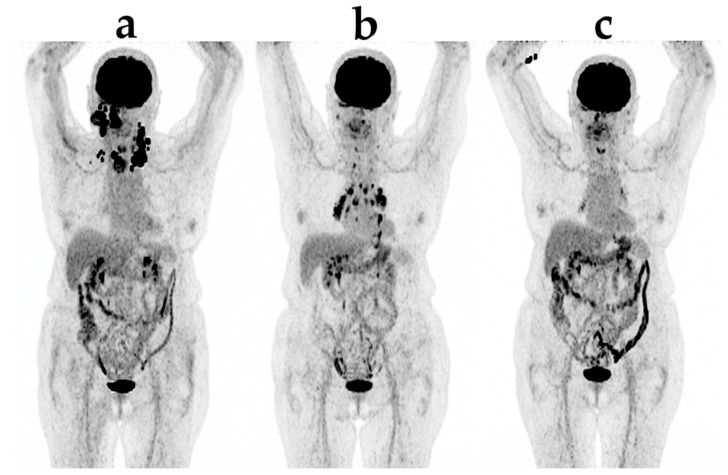
An example of new lesions appearing in an otherwise responding patient due to sarcoid-like irAE (immunerelated adverse event): (**a**) before treatment; (**b**) after three series of pembrolizumab; (**c**) after six series of pembrolizumab.

Using metabolic response evaluation with FDG PET, no formal criteria exists for ICT. Increased tumor burden, it being in FDG-uptake (SUV) or a number of FDG-avid lesions, is usually classified as progression according to the PERCIST and EORTC criteria, both developed for evaluation during chemotherapy [39]. However, care must be taken when new lesions appear in an otherwise responding patient as this could also be due to irAE (Figure 2), e.g., when seen in the adrenals, bowel or pituitary [40].

## 5. New Imaging Biomarkers

To recapitulate, the use of standard imaging methods, anatomical as well as functional, for prediction and early response evaluation during ICT, is hampered by pseudoprogression and late response. Thus, in the scenario of ICT, there is an urgent need for more specific imaging biomarkers, enabling non-invasive, whole-body imaging and potentially improving response evaluation and prediction.

### 5.1. Imaging PD-L1 (Programmed Cell Death Ligand 1)

PD-L1 expression varies greatly between different tumor types, and PD-L1 expression by tumor cells is significantly correlated with objective response and clinical benefit. In metastatic melanoma patients, it was found that patients with PD-L1 positive tumors had a median progression-free survival of 14.0 months and those with PD-L1 negative tumors had a median progression-free survival of 5.3 months, when treated with nivolumab [41]. A study involving melanoma patients treated with pembrolizumab found that PD-L1 expression was significantly associated with both progression free survival and response rate [42]. That PD-L1 could be a general prognostic marker was recently dismissed by a study in tumor samples from 982 patients collected from three trials of adjuvant therapy in early stage NSCLC, finding that PD-L1 expression was not predictive for survival benefit for adjuvant chemotherapy [43].

A number of radiotracers for non-invasive detection of PD-L1 expression have been tested in preclinical models. Maute and colleagues have developed a high affinity non-antibody based PD-1 ectodomain radiolabeled with ^64^Cu, showing affinity for both mouse and human PD-L1 [44]. Being a competitive antagonist of PD-L1, it was used to distinguish between PD-L1 positive and negative tumors in a syngeneic CT26 tumor model. This non-antibody based PD-L1 imaging tracer has increased renal loss compared to antibody based imaging tracer, but does not have the same problems penetrating large tumors as antibodies have [45]. Using a PD-L1 PET imaging agent, it was shown that the lungs of murine models had a particularly strong increase in the expression of PD-L1 when exposed to pro-inflammatory cytokine, giving insights into the development of pneumonitis in anti-PD-L1 therapy [46]. Another PD-L1 PET imaging agent allowed tumor PD-L1 expression to be measured just one hour after injection, making PD-L1 imaging more practical in a clinical setting [47].

SPECT imaging agents have also been developed for imaging PD-L1 biodistribution in vivo [48]. One such agent, a human and mouse cross-reactive antibody radiolabeled with ^111^In, showed a binding of tracer to tumor cells correlating with PD-L1 expression [49]. The study also found that predosing with unlabeled antibody increased tumor uptake, supporting the theory of tissues with natural PD-L1 expression, such as splenocytes, functions as an antigen sink. A similar antibody based SPECT imaging tracer was developed and tested in a murine model of breast cancer, confirming that predosing with unlabeled antibody reduces uptake of the imaging agent in the spleen, increasing concentration of the tracer in the blood stream [50].

### 5.2. ^18^F-Fluorothymidine PET

Another PET tracer that might be of interest in evaluating tumor response to ICT is 3′-deoxy-3′-^18^F-fluorothymidine (FLT). It provides a measure of cellular thymidine kinase 1 activity, which is correlated with cell proliferation [51]. However, in a study with 12 melanoma patients receiving tremelimumab, no significant changes in SUVmax were found between pre- and posttreatment FLT PET-scans [52]. An increase in SUVmax in the spleen was demonstrated, which is consistent with the expected lymphoproliferative effects of the CTLA-4 inhibitor.

### 5.3. T-Cell Tracking

An alternative method for imaging response to ICT is tracking the T-cell response. Anti-CD8 immuno-PET imaging agents would be able to provide information about localization and proliferation of CD8^+^ cell activity. T-cell activation and redistribution to tumor sites happens before any effect of treatment is seen on conventional anatomical imaging. Thus, tracking the T-cell response might provide information about the efficacy of treatment earlier than other image modalities. In one study, antibody fragments radiolabeled with ^64^Cu targeting murine CD8^+^ T-cells, showed specific uptake in the spleen and lymph nodes of antigen-positive mice [53]. A difficulty in using this radiotracer is the abundance of naturally expressed CD8 antigen in some organs, causing the radiotracer to rapidly leave the bloodstream and accumulate in these organs, thereby not reaching other areas of interest such as a tumor [53].

Another radiotracer with specificity for CD8^+^ T-cells, ^89^Zr-desferrioxamine–labeled anti-CD8 cys-diabody, demonstrated the ability to detect changes in tumor infiltrating CD8^+^ T-cell expression in immunocompetent murine syngeneic tumor immunotherapy models receiving anti-PD-L1 antibodies [54]. In this study, responders to treatment showed higher tumor uptake than non-responders, with more intratumoral uptake in responders and a peripheral rim of activity in non-responders. However, for these imaging probes to be validated for potential human use, humanization of the CD8 antibodies is required. Furthermore, where significantly more CD8^+^ cells have been found in responders to most ICTs, no such correlation has been found in patients receiving nivolumab, but more studies are required to confirm these results [55,56].

An ongoing trial is investigating the PET tracer ^18^F-FB-IL2 (^18^F-(fluorobenzoyl)interleukin-2), a tracer with high affinity for the receptor interleukin-2, that is expressed on the surface of T-cells. Using this PET-tracer, the T-cell response to treatment with ipilimumab, nivolumab or pembrolizumab will be examined in human subjects [57].

## 6. Discussion

ICTs have proven effective in treating a number of difficult to treat types of cancer by blocking PD-L1/PD-1 and CTLA-4 checkpoint pathways. Owing to the unconventional method in which these drugs exert their anticancer effect new response patterns are seen, causing evaluation based on CT imaging assessed by standard response criteria such as RECIST to wrongly classify some patients as having treatment failure.

New response criteria for ICT have been proposed, requiring confirmation at a later scan, if the patient is found to have progressed. These novel criteria are to the benefit of the patients who have a delayed response, avoiding discontinuation of effective treatment, but also resulting in treatment being continued in patients for whom treatment has already failed. This can potentially cause a delay in administration of alternative treatment, unnecessary adverse events and costs.

Several studies have demonstrated that patients who were previously classified as having PD per RECIST criteria but non-PD per irRC have higher two-year survival than patients who have PD per both criteria, giving credence to these new response assessment strategies. How this should translate to clinical practice is not yet clear. Guidelines suggest caution has to be taken in discontinuing treatment early until later scans confirm progressive disease [27].

As an alternative to anatomical based criteria, PET could provide functional and perhaps earlier information about the disease and response to treatment. One of the most used PET imaging radiotracers, ^18^F-FDG, seems to provide fairly accurate early response prediction in patients receiving ICT, although similar problems exist as to those with CT, where late responders are classified as having progressive disease. Comparative studies in larger patient samples are, however, urgently needed, before any conclusions can be drawn as to how FDG PET compares to CT in treatment response evaluation in these patients.

Other methods of molecular imaging are currently under development, potentially enabling more specific information about the immune response to the treatment and aid in patient selection, e.g., CD8 and PD-L1 imaging agents. Tracking CD8^+^ cells has shown that a difference in the immune response can be measured between responders and non-responders with PET imaging. This has also provided further insight to how T cells infiltrate tumors in responders and non-responders. If humanization of such radiotracers can be achieved, it can potentially improve our understanding of the response patterns observed with structural imaging and FDG-PET. With PD-L1 expression being upregulated in the tumor environment and its expression shown to be higher in responders than non-responders, imaging the expression of this receptor could help with selecting patients for treatment.

Several radiotracers have been developed for imaging PD-L1 expression, and some of them are also reactive to human PD-L1, thereby opening the possibility for testing these agents in human subjects, as is currently done in on-going clinical trials [58,59].

For both of these types of agents, the specificity for the target receptor has been classified in vitro and the diagnostic accuracy has been determined in murine models. Further research is, however, needed before the efficacy of these imaging agents can be determined [60].

## 7. Conclusions

The use of molecular imaging for response prediction and evaluation during ICT is in its early phase. ^18^F-FDG PET/CT could potentially be useful for early response evaluation; however, the current amount of data is scarce, and clinical trials comparing CT and PET/CT are urgently needed. Using ^18^F-FDG PET/CT for response monitoring does not, however, seem to avoid the pitfall of pseudoprogression.

Novel imaging biomarkers that more specifically address immune activation are under development. These new tracers can potentially enable not only response evaluation, but also a prediction of response to ICT.

## Figures and Tables

**Table 1:** FDG PET/CT ^1^ for response monitoring in immunotherapies.

Study	No. of Patients	Method of Response Assesment	Results
Sachpekidis et al. [32]	22	FDG PET/CT at baseline, after two cycles of ipilimumab and post-treatment. EORTC ^2^ criteria used for response classification	Early scan predictive of post-treatment response in 18 of 22 patients
Kong et al. [33]	27	FDG PET/CT after at least 12 months of treatment with pembrolizumab or nivolumab categorized as positive or negative for presence of metabolically active disease compared to response on CT at the time of the PET/CT scan	43% of patients with residual disease on CT had negative PET scans
Breki et al. [34]	31	FDG PET/CT at baseline, after two cycles of ipilimumab and post-treatment. Fractal and multifractal analysis compared to visual image assesment by nuclear medicine physicians. Seven patients excluded in comparison because of hypermetabolic lesions not related to melanoma (such as irAEs ^3^)	Fractal analysis results match treatment outcome in 20 out of 24 cases
Zheng et al. (Abstract) [35]	28	Retrospective study. FDG PET/CT at baseline and after 2–4 cycles of ipilimumab treatment. Response assessed according to PERCIST ^4^	Two-year survival rate 31% with PMD ^5^ and 73% with non-PMD
Fredrickson et al. (Abstract) [36]	103	Retrospective study. FDG PET/CT at baseline and after six weeks of atezolizumab treatment evaluated according to EORTC	Metabolic responders had higher overall response rate than non-responders (73.9% vs. 6.3%)

^1^ Positron Emission Tomography/Computer Tomography with ^18^F-Fluorodeoxyglucose; ^2^ European Organisation for Research and Treatment of Cancer; ^3^ Immune-related adverse events; ^4^ PET Response Criteria In Solid Tumors; ^5^ Progressive Metabolic Disease.
